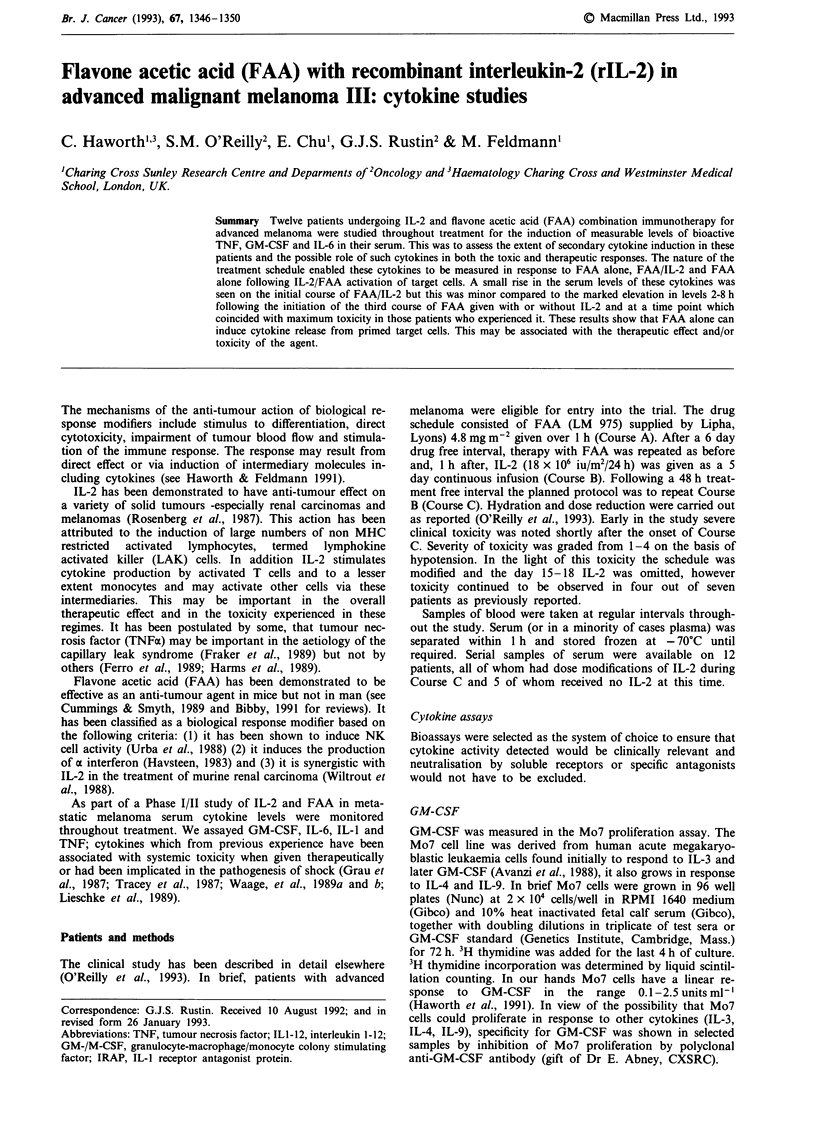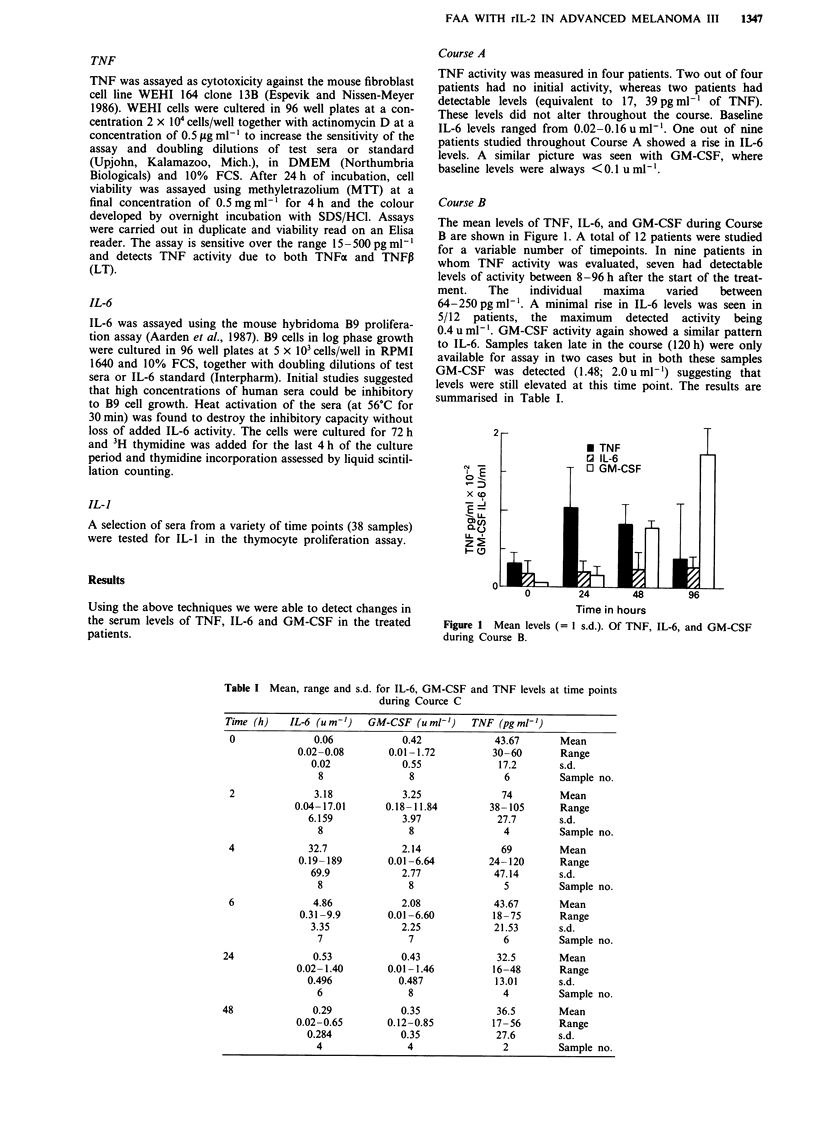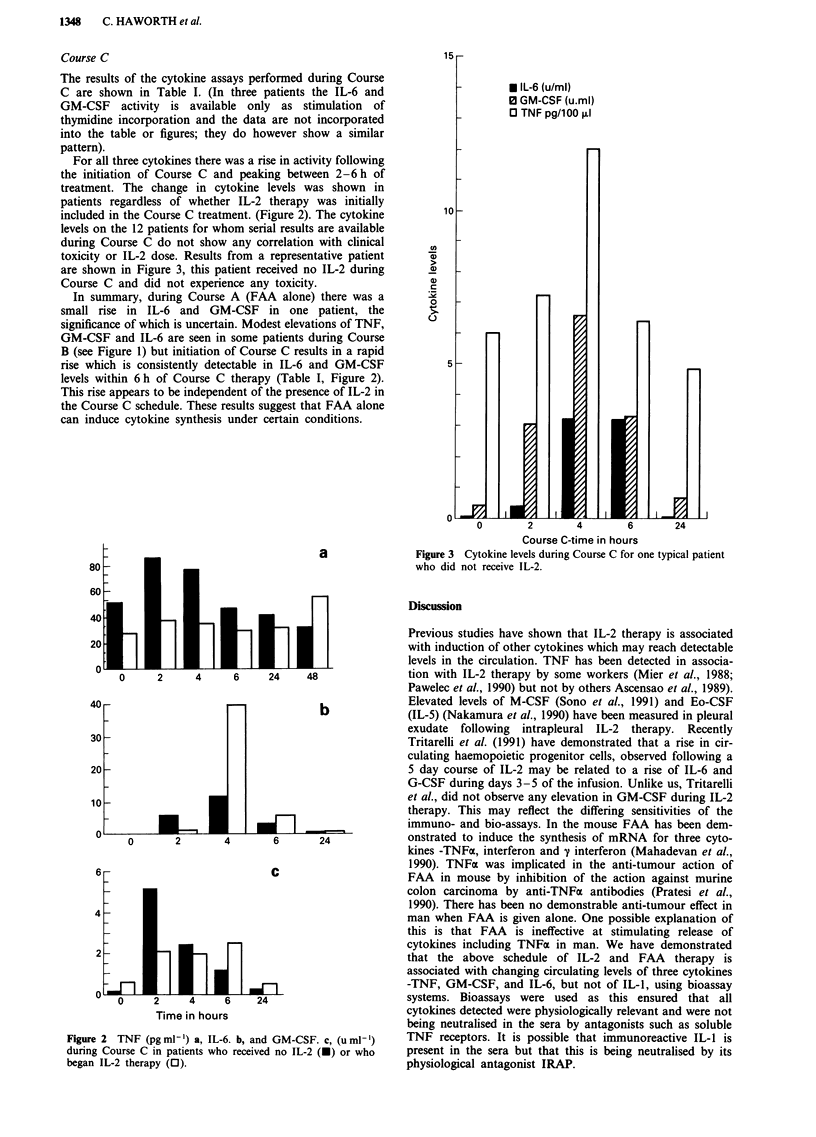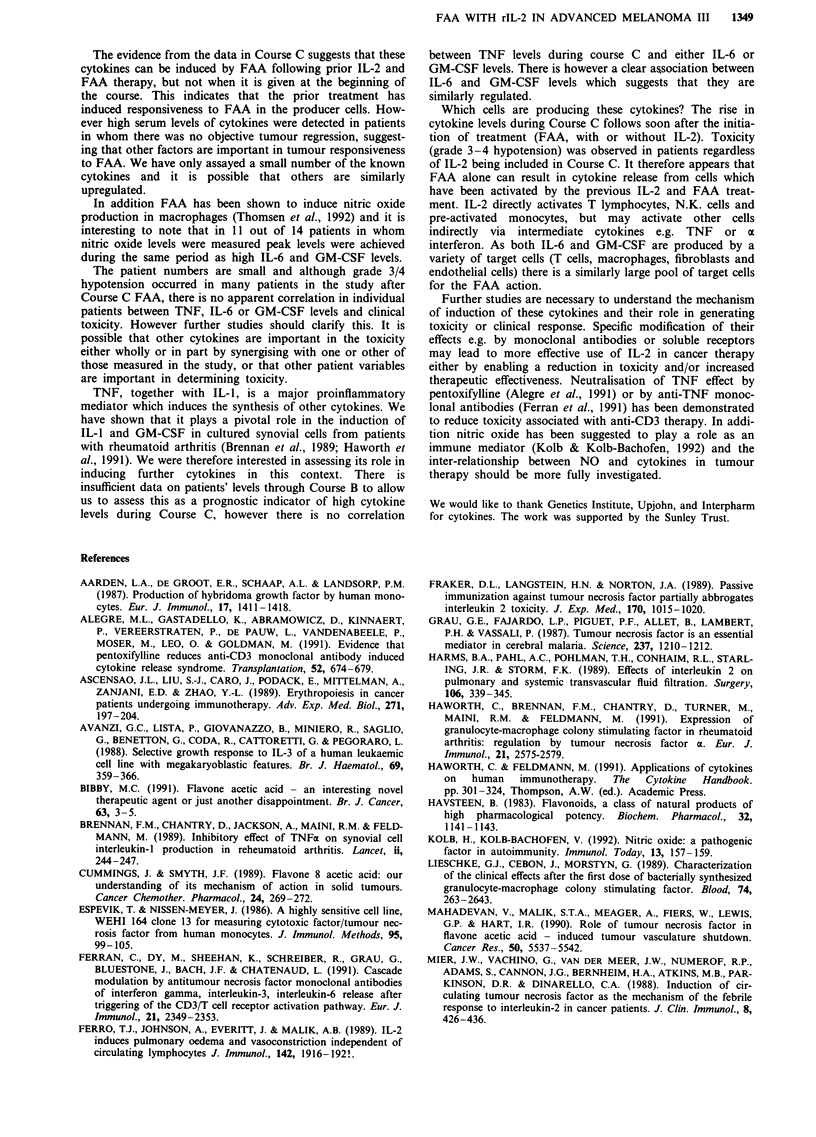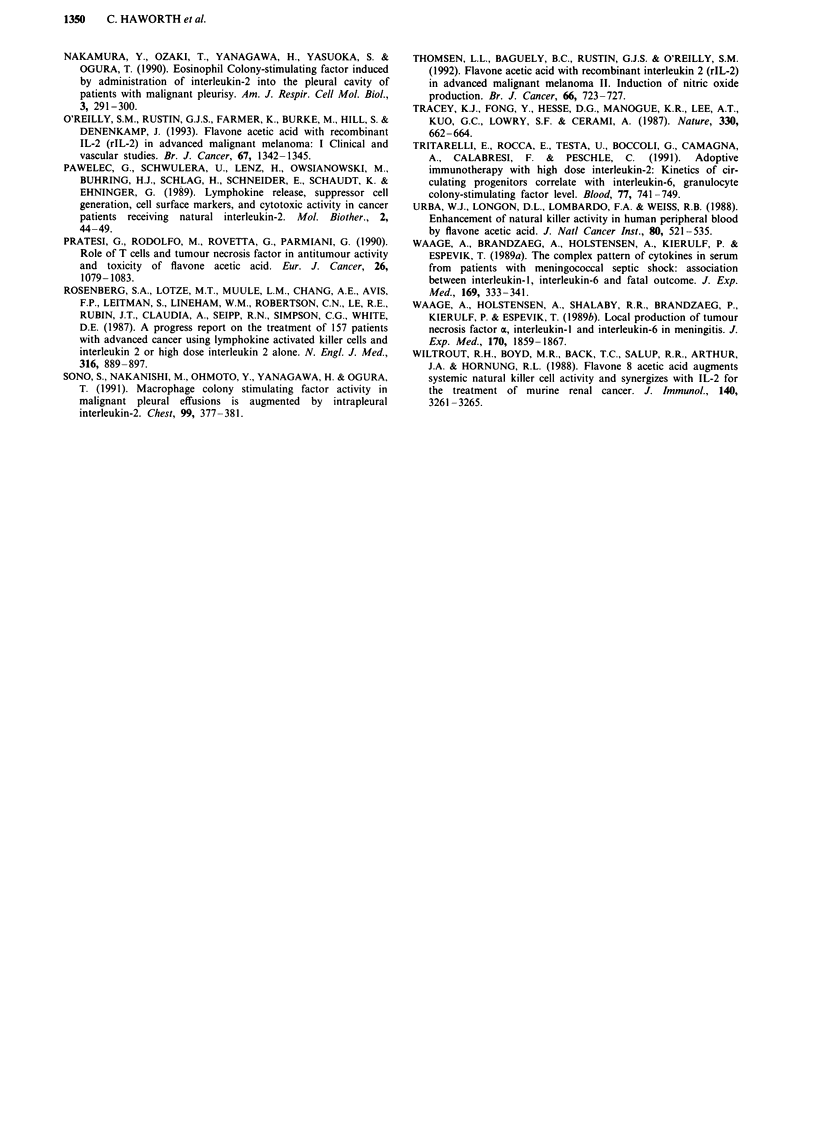# Flavone acetic acid (FAA) with recombinant interleukin-2 (rIL-2) in advanced malignant melanoma. III: Cytokine studies.

**DOI:** 10.1038/bjc.1993.249

**Published:** 1993-06

**Authors:** C. Haworth, S. M. O'Reilly, E. Chu, G. J. Rustin, M. Feldmann

**Affiliations:** Charing Cross Sunley Research Centre, London, UK.

## Abstract

Twelve patients undergoing IL-2 and flavone acetic acid (FAA) combination immunotherapy for advanced melanoma were studied throughout treatment for the induction of measurable levels of bioactive TNF, GM-CSF and IL-6 in their serum. This was to assess the extent of secondary cytokine induction in these patients and the possible role of such cytokines in both the toxic and therapeutic responses. The nature of the treatment schedule enabled these cytokines to be measured in response to FAA alone, FAA/IL-2 and FAA alone following IL-2/FAA activation of target cells. A small rise in the serum levels of these cytokines was seen on the initial course of FAA/IL-2 but this was minor compared to the marked elevation in levels 2-8 h following the initiation of the third course of FAA given with or without IL-2 and at a time point which coincided with maximum toxicity in those patients who experienced it. These results show that FAA alone can induce cytokine release from primed target cells. This may be associated with the therapeutic effect and/or toxicity of the agent.


 © Macmillan Press Ltd., 1993

### Flavone acetic acid (FAA) with recombinant interleukin-2 (rIL-2) in advanced malignant melanoma III: cytokine studies

C. Haworth[1,3], S.M. O'Reilly[2], E. Chu[1], G.J.S. Rustin[2] & M. Feldmann[1]

[1]*Charing Cross Sunley Research Centre and Deparments of* [2]*Oncology and* [3]*Haematology Charing Cross and Westminster Medical School, London, UK.*

**Summary** Twelve patients undergoing IL-2 and flavone acetic acid (FAA) combination immunotherapy for advanced melanoma were studied throughout treatment for the induction of measurable levels of bioactive TNF, GM-CSF and IL-6 in their serum. This was to assess the extent of secondary cytokine induction in these patients and the possible role of such cytokines in both the toxic and therapeutic responses. The nature of the treatment schedule enabled these cytokines to be measured in response to FAA alone, FAA/IL-2 and FAA alone following IL-2/FAA activation of target cells. A small rise in the serum levels of these cytokines was seen on the initial course of FAA/IL-2 but this was minor compared to the marked elevation in levels 2-8 h following the initiation of the third course of FAA given with or without IL-2 and at a time point which coincided with maximum toxicity in those patients who experienced it. These results show that FAA alone can induce cytokine release from primed target cells. This may be associated with the therapeutic effect and/or toxicity of the agent.

The mechanisms of the anti-tumour action of biological response modifiers include stimulus to differentiation, direct cytotoxicity, impairment of tumour blood flow and stimulation of the immune response. The response may result from direct effect or via induction of intermediary molecules including cytokines (see Haworth & Feldmann 1991).

IL-2 has been demonstrated to have anti-tumour effect on a variety of solid tumours -especially renal carcinomas and melanomas (Rosenberg *et al.*, 1987). This action has been attributed to the induction of large numbers of non MHC restricted activated lymphocytes, termed lymphokine activated killer (LAK) cells. In addition IL-2 stimulates cytokine production by activated T cells and to a lesser extent monocytes and may activate other cells via these intermediaries. This may be important in the overall therapeutic effect and in the toxicity experienced in these regimes. It has been postulated by some, that tumour necrosis factor (TNFα) may be important in the aetiology of the capillary leak syndrome (Fraker *et al.*, 1989) but not by others (Ferro *et al.*, 1989; Harms *et al.*, 1989).

Flavone acetic acid (FAA) has been demonstrated to be effective as an anti-tumour agent in mice but not in man (see Cummings & Smyth, 1989 and Bibby, 1991 for reviews). It has been classified as a biological response modifier based on the following criteria: (1) it has been shown to induce NK cell activity (Urba *et al.*, 1988) (2) it induces the production of α interferon (Havsteen, 1983) and (3) it is synergistic with IL-2 in the treatment of murine renal carcinoma (Wiltrout *et al.*, 1988).

As part of a Phase I/II study of IL-2 and FAA in metastatic melanoma serum cytokine levels were monitored throughout treatment. We assayed GM-CSF, IL-6, IL-1 and TNF; cytokines which from previous experience have been associated with systemic toxicity when given therapeutically or had been implicated in the pathogenesis of shock (Grau *et al.*, 1987; Tracey *et al.*, 1987; Waage, *et al.*, 1989*a* and *b*; Lieschke *et al.*, 1989).

### Patients and methods

The clinical study has been described in detail elsewhere (O'Reilly *et al.*, 1993). In brief, patients with advanced melanoma were eligible for entry into the trial. The drug schedule consisted of FAA (LM 975) supplied by Lipha, Lyons) 4.8 mg $m^{-2}$ given over 1 h (Course A). After a 6 day drug free interval, therapy with FAA was repeated as before and, 1 h after, IL-2 ($18 \times 10^6$ iu/$m^2$/24 h) was given as a 5 day continuous infusion (Course B). Following a 48 h treatment free interval the planned protocol was to repeat Course B (Course C). Hydration and dose reduction were carried out as reported (O'Reilly *et al.*, 1993). Early in the study severe clinical toxicity was noted shortly after the onset of Course C. Severity of toxicity was graded from 1–4 on the basis of hypotension. In the light of this toxicity the schedule was modified and the day 15–18 IL-2 was omitted, however toxicity continued to be observed in four out of seven patients as previously reported.

Samples of blood were taken at regular intervals throughout the study. Serum (or in a minority of cases plasma) was separated within 1 h and stored frozen at −70°C until required. Serial samples of serum were available on 12 patients, all of whom had dose modifications of IL-2 during Course C and 5 of whom received no IL-2 at this time.

### *Cytokine assays*

Bioassays were selected as the system of choice to ensure that cytokine activity detected would be clinically relevant and neutralisation by soluble receptors or specific antagonists would not have to be excluded.

### *GM-CSF*

GM-CSF was measured in the Mo7 proliferation assay. The Mo7 cell line was derived from human acute megakaryoblastic leukaemia cells found initially to respond to IL-3 and later GM-CSF (Avanzi *et al.*, 1988), it also grows in response to IL-4 and IL-9. In brief Mo7 cells were grown in 96 well plates (Nunc) at $2 \times 10^4$ cells/well in RPMI 1640 medium (Gibco) and 10% heat inactivated fetal calf serum (Gibco), together with doubling dilutions in triplicate of test sera or GM-CSF standard (Genetics Institute, Cambridge, Mass.) for 72 h. $^3$H thymidine was added for the last 4 h of culture. $^3$H thymidine incorporation was determined by liquid scintillation counting. In our hands Mo7 cells have a linear response to GM-CSF in the range 0.1–2.5 units $ml^{-1}$ (Haworth *et al.*, 1991). In view of the possibility that Mo7 cells could proliferate in response to other cytokines (IL-3, IL-4, IL-9), specificity for GM-CSF was shown in selected samples by inhibition of Mo7 proliferation by polyclonal anti-GM-CSF antibody (gift of Dr E. Abney, CXSRC).

Correspondence: G.J.S. Rustin. Received 10 August 1992; and in revised form 26 January 1993.

Abbreviations: TNF, tumour necrosis factor; IL1-12, interleukin 1-12; GM-/M-CSF, granulocyte-macrophage/monocyte colony stimulating factor; IRAP, IL-1 receptor antagonist protein.

*TNF*

TNF was assayed as cytotoxicity against the mouse fibroblast cell line WEHI 164 clone 13B (Espevik and Nissen-Meyer 1986). WEHI cells were cultered in 96 well plates at a concentration $2 \times 10^4$ cells/well together with actinomycin D at a concentration of 0.5 $\mu g\,ml^{-1}$ to increase the sensitivity of the assay and doubling dilutions of test sera or standard (Upjohn, Kalamazoo, Mich.), in DMEM (Northumbria Biologicals) and 10% FCS. After 24 h of incubation, cell viability was assayed using methyletrazolium (MTT) at a final concentration of 0.5 $mg\,ml^{-1}$ for 4 h and the colour developed by overnight incubation with SDS/HCl. Assays were carried out in duplicate and viability read on an Elisa reader. The assay is sensitive over the range 15–500 $pg\,ml^{-1}$ and detects TNF activity due to both TNF$\alpha$ and TNF$\beta$ (LT).

*IL-6*

IL-6 was assayed using the mouse hybridoma B9 proliferation assay (Aarden *et al*., 1987). B9 cells in log phase growth were cultured in 96 well plates at $5 \times 10^3$ cells/well in RPMI 1640 and 10% FCS, together with doubling dilutions of test sera or IL-6 standard (Interpharm). Initial studies suggested that high concentrations of human sera could be inhibitory to B9 cell growth. Heat activation of the sera (at 56°C for 30 min) was found to destroy the inhibitory capacity without loss of added IL-6 activity. The cells were cultured for 72 h and $^3H$ thymidine was added for the last 4 h of the culture period and thymidine incorporation assessed by liquid scintillation counting.

*IL-1*

A selection of sera from a variety of time points (38 samples) were tested for IL-1 in the thymocyte proliferation assay.

**Results**

Using the above techniques we were able to detect changes in the serum levels of TNF, IL-6 and GM-CSF in the treated patients.

*Course A*

TNF activity was measured in four patients. Two out of four patients had no initial activity, whereas two patients had detectable levels (equivalent to 17, 39 $pg\,ml^{-1}$ of TNF). These levels did not alter throughout the course. Baseline IL-6 levels ranged from 0.02–0.16 $u\,ml^{-1}$. One out of nine patients studied throughout Course A showed a rise in IL-6 levels. A similar picture was seen with GM-CSF, where baseline levels were always $<0.1\ u\,ml^{-1}$.

*Course B*

The mean levels of TNF, IL-6, and GM-CSF during Course B are shown in Figure 1. A total of 12 patients were studied for a variable number of timepoints. In nine patients in whom TNF activity was evaluated, seven had detectable levels of activity between 8–96 h after the start of the treatment. The individual maxima varied between 64–250 $pg\,ml^{-1}$. A minimal rise in IL-6 levels was seen in 5/12 patients, the maximum detected activity being 0.4 $u\,ml^{-1}$. GM-CSF activity again showed a similar pattern to IL-6. Samples taken late in the course (120 h) were only available for assay in two cases but in both these samples GM-CSF was detected (1.48; 2.0 $u\,ml^{-1}$) suggesting that levels were still elevated at this time point. The results are summarised in Table I.

**Figure 1** Mean levels (= 1 s.d.). Of TNF, IL-6, and GM-CSF during Course B.

**Table I** Mean, range and s.d. for IL-6, GM-CSF and TNF levels at time points during Cource C

| *Time (h)* | *IL-6 ($u\,m^{-1}$)* | *GM-CSF ($u\,ml^{-1}$)* | *TNF ($pg\,ml^{-1}$)* | |
|---|---|---|---|---|
| 0 | 0.06 | 0.42 | 43.67 | Mean |
| | 0.02–0.08 | 0.01–1.72 | 30–60 | Range |
| | 0.02 | 0.55 | 17.2 | s.d. |
| | 8 | 8 | 6 | Sample no. |
| 2 | 3.18 | 3.25 | 74 | Mean |
| | 0.04–17.01 | 0.18–11.84 | 38–105 | Range |
| | 6.159 | 3.97 | 27.7 | s.d. |
| | 8 | 8 | 4 | Sample no. |
| 4 | 32.7 | 2.14 | 69 | Mean |
| | 0.19–189 | 0.01–6.64 | 24–120 | Range |
| | 69.9 | 2.77 | 47.14 | s.d. |
| | 8 | 8 | 5 | Sample no. |
| 6 | 4.86 | 2.08 | 43.67 | Mean |
| | 0.31–9.9 | 0.01–6.60 | 18–75 | Range |
| | 3.35 | 2.25 | 21.53 | s.d. |
| | 7 | 7 | 6 | Sample no. |
| 24 | 0.53 | 0.43 | 32.5 | Mean |
| | 0.02–1.40 | 0.01–1.46 | 16–48 | Range |
| | 0.496 | 0.487 | 13.01 | s.d. |
| | 6 | 8 | 4 | Sample no. |
| 48 | 0.29 | 0.35 | 36.5 | Mean |
| | 0.02–0.65 | 0.12–0.85 | 17–56 | Range |
| | 0.284 | 0.35 | 27.6 | s.d. |
| | 4 | 4 | 2 | Sample no. |

*Course C*

The results of the cytokine assays performed during Course C are shown in Table I. (In three patients the IL-6 and GM-CSF activity is available only as stimulation of thymidine incorporation and the data are not incorporated into the table or figures; they do however show a similar pattern).

For all three cytokines there was a rise in activity following the initiation of Course C and peaking between 2–6 h of treatment. The change in cytokine levels was shown in patients regardless of whether IL-2 therapy was initially included in the Course C treatment. (Figure 2). The cytokine levels on the 12 patients for whom serial results are available during Course C do not show any correlation with clinical toxicity or IL-2 dose. Results from a representative patient are shown in Figure 3, this patient received no IL-2 during Course C and did not experience any toxicity.

In summary, during Course A (FAA alone) there was a small rise in IL-6 and GM-CSF in one patient, the significance of which is uncertain. Modest elevations of TNF, GM-CSF and IL-6 are seen in some patients during Course B (see Figure 1) but initiation of Course C results in a rapid rise which is consistently detectable in IL-6 and GM-CSF levels within 6 h of Course C therapy (Table I, Figure 2). This rise appears to be independent of the presence of IL-2 in the Course C schedule. These results suggest that FAA alone can induce cytokine synthesis under certain conditions.

**Figure 2** TNF (pg $ml^{-1}$) **a**, IL-6. **b**, and GM-CSF. **c**, (u $ml^{-1}$) during Course C in patients who received no IL-2 (■) or who began IL-2 therapy (□).

**Figure 3** Cytokine levels during Course C for one typical patient who did not receive IL-2.

### Discussion

Previous studies have shown that IL-2 therapy is associated with induction of other cytokines which may reach detectable levels in the circulation. TNF has been detected in association with IL-2 therapy by some workers (Mier *et al.*, 1988; Pawelec *et al.*, 1990) but not by others Ascensao *et al.*, 1989). Elevated levels of M-CSF (Sono *et al.*, 1991) and Eo-CSF (IL-5) (Nakamura *et al.*, 1990) have been measured in pleural exudate following intrapleural IL-2 therapy. Recently Tritarelli *et al.* (1991) have demonstrated that a rise in circulating haemopoietic progenitor cells, observed following a 5 day course of IL-2 may be related to a rise of IL-6 and G-CSF during days 3–5 of the infusion. Unlike us, Tritarelli *et al.*, did not observe any elevation in GM-CSF during IL-2 therapy. This may reflect the differing sensitivities of the immuno- and bio-assays. In the mouse FAA has been demonstrated to induce the synthesis of mRNA for three cytokines -TNFα, interferon and γ interferon (Mahadevan *et al.*, 1990). TNFα was implicated in the anti-tumour action of FAA in mouse by inhibition of the action against murine colon carcinoma by anti-TNFα antibodies (Pratesi *et al.*, 1990). There has been no demonstrable anti-tumour effect in man when FAA is given alone. One possible explanation of this is that FAA is ineffective at stimulating release of cytokines including TNFα in man. We have demonstrated that the above schedule of IL-2 and FAA therapy is associated with changing circulating levels of three cytokines -TNF, GM-CSF, and IL-6, but not of IL-1, using bioassay systems. Bioassays were used as this ensured that all cytokines detected were physiologically relevant and were not being neutralised in the sera by antagonists such as soluble TNF receptors. It is possible that immunoreactive IL-1 is present in the sera but that this is being neutralised by its physiological antagonist IRAP.

The evidence from the data in Course C suggests that these cytokines can be induced by FAA following prior IL-2 and FAA therapy, but not when it is given at the beginning of the course. This indicates that the prior treatment has induced responsiveness to FAA in the producer cells. However high serum levels of cytokines were detected in patients in whom there was no objective tumour regression, suggesting that other factors are important in tumour responsiveness to FAA. We have only assayed a small number of the known cytokines and it is possible that others are similarly upregulated.

In addition FAA has been shown to induce nitric oxide production in macrophages (Thomsen *et al.*, 1992) and it is interesting to note that in 11 out of 14 patients in whom nitric oxide levels were measured peak levels were achieved during the same period as high IL-6 and GM-CSF levels.

The patient numbers are small and although grade 3/4 hypotension occurred in many patients in the study after Course C FAA, there is no apparent correlation in individual patients between TNF, IL-6 or GM-CSF levels and clinical toxicity. However further studies should clarify this. It is possible that other cytokines are important in the toxicity either wholly or in part by synergising with one or other of those measured in the study, or that other patient variables are important in determining toxicity.

TNF, together with IL-1, is a major proinflammatory mediator which induces the synthesis of other cytokines. We have shown that it plays a pivotal role in the induction of IL-1 and GM-CSF in cultured synovial cells from patients with rheumatoid arthritis (Brennan *et al.*, 1989; Haworth *et al.*, 1991). We were therefore interested in assessing its role in inducing further cytokines in this context. There is insufficient data on patients' levels through Course B to allow us to assess this as a prognostic indicator of high cytokine levels during Course C, however there is no correlation between TNF levels during course C and either IL-6 or GM-CSF levels. There is however a clear association between IL-6 and GM-CSF levels which suggests that they are similarly regulated.

Which cells are producing these cytokines? The rise in cytokine levels during Course C follows soon after the initiation of treatment (FAA, with or without IL-2). Toxicity (grade 3–4 hypotension) was observed in patients regardless of IL-2 being included in Course C. It therefore appears that FAA alone can result in cytokine release from cells which have been activated by the previous IL-2 and FAA treatment. IL-2 directly activates T lymphocytes, N.K. cells and pre-activated monocytes, but may activate other cells indirectly via intermediate cytokines e.g. TNF or α interferon. As both IL-6 and GM-CSF are produced by a variety of target cells (T cells, macrophages, fibroblasts and endothelial cells) there is a similarly large pool of target cells for the FAA action.

Further studies are necessary to understand the mechanism of induction of these cytokines and their role in generating toxicity or clinical response. Specific modification of their effects e.g. by monoclonal antibodies or soluble receptors may lead to more effective use of IL-2 in cancer therapy either by enabling a reduction in toxicity and/or increased therapeutic effectiveness. Neutralisation of TNF effect by pentoxifylline (Alegre *et al.*, 1991) or by anti-TNF monoclonal antibodies (Ferran *et al.*, 1991) has been demonstrated to reduce toxicity associated with anti-CD3 therapy. In addition nitric oxide has been suggested to play a role as an immune mediator (Kolb & Kolb-Bachofen, 1992) and the inter-relationship between NO and cytokines in tumour therapy should be more fully investigated.

We would like to thank Genetics Institute, Upjohn, and Interpharm for cytokines. The work was supported by the Sunley Trust.

### References

AARDEN, L.A., DE GROOT, E.R., SCHAAP, A.L. & LANDSORP, P.M. (1987). Production of hybridoma growth factor by human monocytes. *Eur. J. Immunol.*, **17**, 1411–1418.

ALEGRE, M.L., GASTADELLO, K., ABRAMOWICZ, D., KINNAERT, P., VEREERSTRATEN, P., DE PAUW, L., VANDENABEELE, P., MOSER, M., LEO, O. & GOLDMAN, M. (1991). Evidence that pentoxifylline reduces anti-CD3 monoclonal antibody induced cytokine release syndrome. *Transplantation*, **52**, 674–679.

ASCENSAO, J.L., LIU, S.-J., CARO, J., PODACK, E., MITTELMAN, A., ZANJANI, E.D. & ZHAO, Y.-L. (1989). Erythropoiesis in cancer patients undergoing immunotherapy. *Adv. Exp. Med. Biol.*, **271**, 197–204.

AVANZI, G.C., LISTA, P., GIOVANAZZO, B., MINIERO, R., SAGLIO, G., BENETTON, G., CODA, R., CATTORETTI, G. & PEGORARO, L. (1988). Selective growth response to IL-3 of a human leukaemic cell line with megakaryoblastic features. *Br. J. Haematol.*, **69**, 359–366.

BIBBY, M.C. (1991). Flavone acetic acid – an interesting novel therapeutic agent or just another disappointment. *Br. J. Cancer*, **63**, 3–5.

BRENNAN, F.M., CHANTRY, D., JACKSON, A., MAINI, R.M. & FELDMANN, M. (1989). Inhibitory effect of TNFα on synovial cell interleukin-1 production in reheumatoid arthritis. *Lancet*, **ii**, 244–247.

CUMMINGS, J. & SMYTH, J.F. (1989). Flavone 8 acetic acid: our understanding of its mechanism of action in solid tumours. *Cancer Chemother. Pharmacol.*, **24**, 269–272.

ESPEVIK, T. & NISSEN-MEYER, J. (1986). A highly sensitive cell line, WEHI 164 clone 13 for measuring cytotoxic factor/tumour necrosis factor from human monocytes. *J. Immunol. Methods*, **95**, 99–105.

FERRAN, C., DY, M., SHEEHAN, K., SCHREIBER, R., GRAU, G., BLUESTONE, J., BACH, J.F. & CHATENAUD, L. (1991). Cascade modulation by antitumour necrosis factor monoclonal antibodies of interferon gamma, interleukin-3, interleukin-6 release after triggering of the CD3/T cell receptor activation pathway. *Eur. J. Immunol.*, **21**, 2349–2353.

FERRO, T.J., JOHNSON, A., EVERITT, J. & MALIK, A.B. (1989). IL-2 induces pulmonary oedema and vasoconstriction independent of circulating lymphocytes *J. Immunol.*, **142**, 1916–1921.

FRAKER, D.L., LANGSTEIN, H.N. & NORTON, J.A. (1989). Passive immunization against tumour necrosis factor partially abbrogates interleukin 2 toxicity. *J. Exp. Med.*, **170**, 1015–1020.

GRAU, G.E., FAJARDO, L.P., PIGUET, P.F., ALLET, B., LAMBERT, P.H. & VASSALI, P. (1987). Tumour necrosis factor is an essential mediator in cerebral malaria. *Science*, **237**, 1210–1212.

HARMS, B.A., PAHL, A.C., POHLMAN, T.H., CONHAIM, R.L., STARLING, J.R. & STORM, F.K. (1989). Effects of interleukin 2 on pulmonary and systemic transvascular fluid filtration. *Surgery*, **106**, 339–345.

HAWORTH, C., BRENNAN, F.M., CHANTRY, D., TURNER, M., MAINI, R.M. & FELDMANN, M. (1991). Expression of granulocyte-macrophage colony stimulating factor in rheumatoid arthritis: regulation by tumour necrosis factor α. *Eur. J. Immunol.*, **21**, 2575-2579.

HAWORTH, C. & FELDMANN, M. (1991). Applications of cytokines on human immunotherapy. *The Cytokine Handbook*. pp. 301–324, Thompson, A.W. (ed.). Academic Press.

HAVSTEEN, B. (1983). Flavonoids, a class of natural products of high pharmacological potency. *Biochem. Pharmacol.*, **32**, 1141–1143.

KOLB, H., KOLB-BACHOFEN, V. (1992). Nitric oxide: a pathogenic factor in autoimmunity. *Immunol. Today*, **13**, 157–159.

LIESCHKE, G.J., CEBON, J., MORSTYN, G. (1989). Characterization of the clinical effects after the first dose of bacterially synthesized granulocyte-macrophage colony stimulating factor. *Blood*, **74**, 263–2643.

MAHADEVAN, V., MALIK, S.T.A., MEAGER, A., FIERS, W., LEWIS, G.P. & HART, I.R. (1990). Role of tumour necrosis factor in flavone acetic acid – induced tumour vasculature shutdown. *Cancer Res.*, **50**, 5537–5542.

MIER, J.W., VACHINO, G., VAN DER MEER, J.W., NUMEROF, R.P., ADAMS, S., CANNON, J.G., BERNHEIM, H.A., ATKINS, M.B., PARKINSON, D.R. & DINARELLO, C.A. (1988). Induction of circulating tumour necrosis factor as the mechanism of the febrile response to interleukin-2 in cancer patients. *J. Clin. Immunol.*, **8**, 426–436.

NAKAMURA, Y., OZAKI, T., YANAGAWA, H., YASUOKA, S. & OGURA, T. (1990). Eosinophil Colony-stimulating factor induced by administration of interleukin-2 into the pleural cavity of patients with malignant pleurisy. *Am. J. Respir. Cell Mol. Biol.*, **3**, 291–300.

O'REILLY, S.M., RUSTIN, G.J.S., FARMER, K., BURKE, M., HILL, S. & DENENKAMP, J. (1993). Flavone acetic acid with recombinant IL-2 (rIL-2) in advanced malignant melanoma: I Clinical and vascular studies. *Br. J. Cancer*, **67**, 1342–1345.

PAWELEC, G., SCHWULERA, U., LENZ, H., OWSIANOWSKI, M., BUHRING, H.J., SCHLAG, H., SCHNEIDER, E., SCHAUDT, K. & EHNINGER, G. (1989). Lymphokine release, suppressor cell generation, cell surface markers, and cytotoxic activity in cancer patients receiving natural interleukin-2. *Mol. Biother.*, **2**, 44–49.

PRATESI, G., RODOLFO, M., ROVETTA, G., PARMIANI, G. (1990). Role of T cells and tumour necrosis factor in antitumour activity and toxicity of flavone acetic acid. *Eur. J. Cancer*, **26**, 1079–1083.

ROSENBERG, S.A., LOTZE, M.T., MUULE, L.M., CHANG, A.E., AVIS, F.P., LEITMAN, S., LINEHAM, W.M., ROBERTSON, C.N., LE, R.E., RUBIN, J.T., CLAUDIA, A., SEIPP, R.N., SIMPSON, C.G., WHITE, D.E. (1987). A progress report on the treatment of 157 patients with advanced cancer using lymphokine activated killer cells and interleukin 2 or high dose interleukin 2 alone. *N. Engl. J. Med.*, **316**, 889–897.

SONO, S., NAKANISHI, M., OHMOTO, Y., YANAGAWA, H. & OGURA, T. (1991). Macrophage colony stimulating factor activity in malignant pleural effusions is augmented by intrapleural interleukin-2. *Chest*, **99**, 377–381.

THOMSEN, L.L., BAGUELY, B.C., RUSTIN, G.J.S. & O'REILLY, S.M. (1992). Flavone acetic acid with recombinant interleukin 2 (rIL-2) in advanced malignant melanoma II. Induction of nitric oxide production. *Br. J. Cancer*, **66**, 723–727.

TRACEY, K.J., FONG, Y., HESSE, D.G., MANOGUE, K.R., LEE, A.T., KUO, G.C., LOWRY, S.F. & CERAMI, A. (1987). *Nature*, **330**, 662–664.

TRITARELLI, E., ROCCA, E., TESTA, U., BOCCOLI, G., CAMAGNA, A., CALABRESI, F. & PESCHLE, C. (1991). Adoptive immunotherapy with high dose interleukin-2: Kinetics of circulating progenitors correlate with interleukin-6, granulocyte colony-stimulating factor level. *Blood*, **77**, 741–749.

URBA, W.J., LONGON, D.L., LOMBARDO, F.A. & WEISS, R.B. (1988). Enhancement of natural killer activity in human peripheral blood by flavone acetic acid. *J. Natl Cancer Inst.*, **80**, 521–535.

WAAGE, A., BRANDZAEG, A., HOLSTENSEN, A., KIERULF, P. & ESPEVIK, T. (1989*a*). The complex pattern of cytokines in serum from patients with meningococcal septic shock: association between interleukin-1, interleukin-6 and fatal outcome. *J. Exp. Med.*, **169**, 333–341.

WAAGE, A., HOLSTENSEN, A., SHALABY, R.R., BRANDZAEG, P., KIERULF, P. & ESPEVIK, T. (1989*b*). Local production of tumour necrosis factor α, interleukin-1 and interleukin-6 in meningitis. *J. Exp. Med.*, **170**, 1859–1867.

WILTROUT, R.H., BOYD, M.R., BACK, T.C., SALUP, R.R., ARTHUR, J.A. & HORNUNG, R.L. (1988). Flavone 8 acetic acid augments systemic natural killer cell activity and synergizes with IL-2 for the treatment of murine renal cancer. *J. Immunol.*, **140**, 3261–3265.